# Examining Different Types of Sleep Among Custodial Grandparents During COVID-19

**DOI:** 10.1093/geroni/igab046.3688

**Published:** 2021-12-17

**Authors:** Karen Clark, Kellie Mayfield, Raeda Anderson

**Affiliations:** 1 Southern University and A&M College, Baton Rouge, Louisiana, United States; 2 Georgia State University Byrdine Lewis College of Nursing and Health, Atlanta, Georgia, United States; 3 Shepherd Center, Atlanta, Georgia, United States

## Abstract

Sleep is associated with healthy living. With increased age, sleep is harder to initiate and maintain. Currently, over two million grandparents have become primary caregivers to their grandchildren and are at risk for poor sleep outcomes. Research shows that grandparent caregivers are at risk for depression due to poor sleep quality. Thus, this study aimed to identify the sleep quality of custodial grandparents to gain a better understanding of sleep patterns during COVID-19 in 2020. Thirty-four custodial grandparents were recruited from the Georgia Division of Aging Kinship Care Support Groups from September through October 2020. Participants were between 42 to 78 years old with a mean age of 57. Participants completed the Pittsburgh Sleep Quality Index. Stata statistical software was used to analyze the relationship between the sleep quality subscales. Results showed a significant positive relationship for custodial grandparents between sleep quality and daytime dysfunction (χ2=25.993, p=0.002; Γ=0.495, p=0.039) as well as sleep quality and sleep disturbance (χ2=11.129, p=0.084; Γ=0.751, p<0.001). There is a significant positive relationship between daytime dysfunction and sleep duration (χ2=14.984, p=0.091; Γ=0.681, p<.001), where grandparents with daytime dysfunction have longer sleep duration. Findings suggest grandparents with poor sleep quality are more likely to experience daytime dysfunction and have more sleep disturbances in the COVID-19 environment. Our study will benefit researchers and practitioners caring for custodial grandparents and contribute to future research focused on custodial grandparents and sleep quality.

